# Emphysematous Pyelonephritis in Cancer Patients: A Case Report With a Literature Review

**DOI:** 10.7759/cureus.65000

**Published:** 2024-07-20

**Authors:** Karandeep Singh, John Greene

**Affiliations:** 1 Internal Medicine, Government Medical College, Amritsar, Amritsar, IND; 2 Infectious Disease, Moffitt Cancer Center and Research Institute, Tampa, USA

**Keywords:** e. coli, diabetes mellitus, cancer, klebsiella, emphysematous pyelonephritis

## Abstract

Emphysematous pyelonephritis (EPN) is a severe necrotising infection of the renal parenchyma which is rarely reported in cancer patients. We present a case of a 92-year-old man with urothelial carcinoma of the bladder who developed EPN after resection of the tumour. Septic shock developed and blood cultures grew extended-spectrum beta-lactamase (ESBL) *Escherichia coli.* Because of renal function decline with hematuria a non-contrast CT scan of the abdomen showed gas in the kidney and surrounding area consistent with EPN. After receiving appropriate antibiotics according to susceptibility testing, EPN was resolved based on a follow-up CT scan of the abdomen without requiring further surgical intervention. We review the literature and discuss the management of EPN in cancer patients.

## Introduction

Emphysematous pyelonephritis (EPN) is a severe necrotizing infection of the renal parenchyma and perinephric tissue which can be fatal if not treated promptly [1]. It is caused by gas-forming gram-negative organisms, such as *Escherichia coli *(*E. coli*) and *Klebsiella* which are the most common. Other examples include *Clostridium*, *Candida*, *Pseudomonas*, *Aspergillus,* etc. [2]. Although the pathogenesis is not well understood, it is believed that the increased glucose content of renal tissue predisposes to EPN, hence, diabetes mellitus is the most common risk factor [3]. EPN occurs more frequently in females than in males [4]. Other risk factors for EPN include immunodeficiency, ureteral obstruction, impaired renal vascular supply, presence of gas-forming organisms, etc. [5]. It is a potentially fatal infection with a mortality rate ranging from 11% to 42% [6]. Treatment includes fluid resuscitation and appropriate antibiotics [4]. Conversely, in cases that are slow to respond to conservative therapy, nephrostomy tube placement and partial or radical nephrectomy can be performed [7]. Because EPN is a rare entity with very few cases reported in cancer patients, we present our case with a review of the literature.

## Case presentation

A 92-year-old man with a past medical history (PMH) of prostate carcinoma status post (s/p) radiation therapy was admitted to our facility for a muscle-invasive bladder tumour, which was high-grade urothelial carcinoma with extensive squamous differentiation and necrosis. He underwent a radical cystoprostatectomy with bilateral pelvic node dissection, ileal conduit urinary diversion with urethral stent placement and resection of a small bowel secondary to local tumour invasion.

On the postoperative day (POD) 5, he went into septic shock with blood pressure (BP) of 89/43, heart rate (HR) of 133, temperature (Temp) of 98.3°F, and respiratory rate (RR) of 31. His white blood cell (WBC) was 13,000/microL and his glucose level was 177 mg/dL. He was transferred to the intensive care unit (ICU) where he received vasopressors. His blood cultures grew multidrug-resistant (MDR) *E. coli,* resistant to ceftriaxone and ciprofloxacin. He was treated with intravenous (IV) meropenem 500mg, thrice daily. His symptoms improved and pressors were stopped after two days.

**Figure 1:**
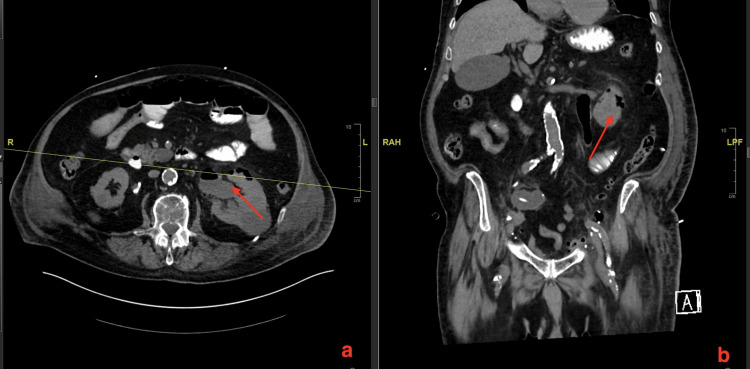
A CT scan of his abdomen pelvis without contrast shows gas in the left renal parenchyma and surrounding perinephric tissue. (a) A CT scan showing the axial section of renal parenchyma and perinephric area with gas. (b) A CT scan showing the coronal section of renal parenchyma and perinephric area with gas.

**Figure 2:**
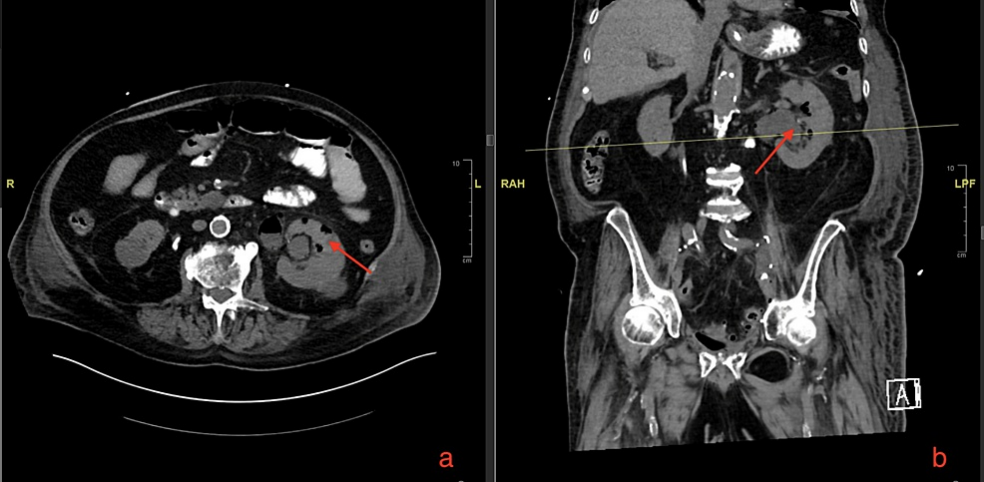
A CT scan of his abdomen pelvis without contrast shows gas in the left renal parenchyma. (a) A CT scan showing the axial section of renal parenchyma with gas. (b) A CT scan showing the coronal section of renal parenchyma with gas.

**Figure 3:**
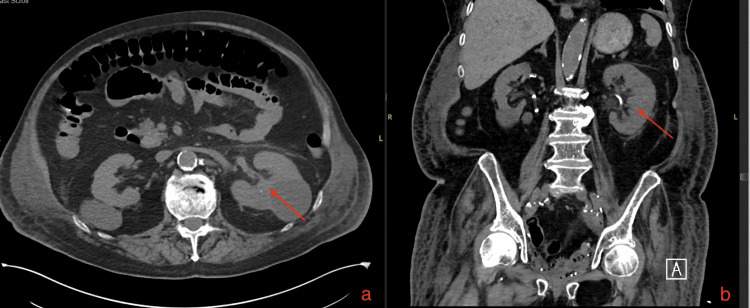
A CT scan of his abdomen pelvis shows clearance of gas and normal left renal parenchyma demarcated by a red arrow. (a) A CT scan of the axial section showing clearance of gas. (b) A CT scan of the coronal section showing clearance of gas.

Meanwhile, renal function became worse. Although the patient's baseline blood urea nitrogen (BUN) was 30 and creatinine (Cr) was 1.0 on admission, on ICU day 2 the BUN was 92, Cr was 2.3 and glucose level was 240 mg/dL. Urine analysis demonstrated hematuria with urine analysis showing more than 11-20 RBC/mL and 11-20 WBC/mL. CT scan of the abdomen and pelvis (Figures 1-2) demonstrated EPN and displacement of the left ureteral stent. Ureteral stent exchange was done the same day along with continuation of meropenem. Subsequent urine culture grew mixed bacterial flora with no further identification. Acute kidney injury (AKI) improved after ureter stent placement with BUN coming down to 40 and Cr to 1.7. CT scan of the abdomen and pelvis five days later showed improvement in pyelonephritis (Figure 3). The ureter stents were removed six days after the second CT. He was discharged on intravenous ertapenem 500mg daily for 10 more days making a total antibiotic course of three weeks.

On POD 18, serous leakage from the wound site developed and a Cr staining of discharge came out to be positive, hence confirming a urine leakage. A loopogram showed leakage of dye directly into the pelvis from the ileal conduit. Percutaneous nephrostomy tube placement was performed followed by internalisation of urine drainage with a ureter stent placement one week later. He was discharged in a stable condition and had no further urine infections. Our patient was prediabetic with a baseline fasting glucose level of 125 mg/dL. He was not taking glucose-lowering medications prior to hospitalisation and was hyperglycemic throughout the hospital stay.

## Discussion

This case describes a rare and severe form of EPN in a cancer patient. It was successfully managed medically without additional surgery. Early diagnosis and appropriate management of EPN are very important as patients are already immunocompromised due to their cancer diagnosis and treatment. The first ever documented case of EPN was described by Kelly and MacCallum in 1898 [8]. The term "emphysematous pyelonephritis" was coined and delineated by Schultz and Klorfein in 1962 [9]. This disease predominantly occurs in females as evidenced by the literature review table summarising all reported cases of EPN in cancer patients (Table 1). It occurs in old age with patients in their 60s-70s and commonly those with diabetes mellitus (Table 1).

**Table 1:** A literature review of reported cases of emphysematous pyelonephritis in cancer patients CA: carcinoma; EPN: emphysematous pyelonephritis; F: female; IV: intravenous

Case	Age	Sex	Type of cancer	DM	Other risk factors	Causative organism	Treatment received	Outcome
Zanon et al., 2023 [10]	64	F	Cervical carcinoma	NO	Abdominal surgery	Klebsiella pneumoniae	Percutaneous nephrostomy and antibiotics	Died after one month
Elvas et al., 2018 [11]	64	F	Urothelial CA bladder	NO	Urothelial cancer-causing obstruction	Sphingomonas (Pseudomonas) paucimobilis	Nephrectomy	Died of hemodynamic instability
Yokoyama et al., 2017 [12]	67	F	Lung CA	YES	Receiving dexamethasone led to deranged sugar levels	K. pneumoniae	IV antibiotics and ureteral stent placement	Survived
Mosholt et al., 2015 [13]	88	F	Renal CA	NO	Obstructing renal tumour	Escherichia coli	IV antibiotics and ureteral stent placement	Survived
Sato et al., 2010 [14]	61	F	Renal and bladder CA	YES	Post nephrectomy EPN	K. pneumoniae	IV antibiotic presented with a shock	Died
Takizawa et al., 2000 [15]	75	F	Renal cell CA	YES	Tumor was obstructing	E. coli	IV antibiotic followed by nephrectomy	Survived

Our patient was pre-diabetic having blood glucose levels of 126 at baseline but at the time of having EPN his blood glucose level was 223 mg/dL. The most common organism causing EPN is *E. coli *followed by *Klebsiella pneumonia*. Other organisms that can cause EPN are *Clostridium perfringens*, *Proteus mirabilis*, *Pseudomonas*, etc. (Table 2) [2]. In our case, extended-spectrum beta-lactamase (ESBL) *E. coli *was cultured in blood on MacConkey agar, while urine culture showed mixed flora. It is postulated that cancer patients, with an immunocompromised state, decreased tissue perfusion and increased glucose in the parenchyma (if the patient is diabetic) are predisposed to EPN [12,16]. This infection is more common in patients with pelvic tumours, including renal cell carcinoma, urothelial cancer of the bladder, prostate cancer, cervical cancer and ovarian cancer. These tumours can create a physical obstruction of the ureter or lower genitourinary tract, resulting in hydronephrosis and increasing susceptibility to the development of pyelonephritis. In our patient, cystoprostatectomy with a urinary conduit could increase vesicoureteral reflux and thus development of pyelonephritis. Imaging studies used to diagnose EPN include plain film X-ray, and ultrasound can show signs of gas in the renal parenchyma but are nonspecific with overshadowing bowel gas as a limiting factor. With ultrasound, renal calculi can also produce an artefact which can be confused with gas [17]. EPN is mostly diagnosed by imaging modalities such as CT scan of the abdomen and pelvis (Figures 1-2). The sensitivity of a CT scan to diagnose EPN is 100% as compared to ultrasound which is 69% and plain X-ray is 65% [18]. MRI scan can also be used in selected cases but CT scan is the imaging modality of choice.

**Table 2:** Microorganisms causing emphysematous pyelonephritis Reference [4]

Causative organism	% of infection
Escherichia coli	49-67%
Klebsiella	20-24%
Proteus	5-18%
Enterococcus	14%
Pseudomonas	5%
Polymicrobial	4-24%

Initial management of patients with EPN is administrating fluid resuscitation and antibiotics, along with blood and urine culture with susceptibility testing [19]. Antibiotic therapy should be given empirically in all patients with EPN infection on the lines of complicated urinary tract infection guidelines, but fluoroquinolones have shown to be limited in efficacy due to increasing resistance [20]. Empiric antibiotic therapy should target all the common organisms such as *E. coli*, *Klebsiella*, and *Proteus* and should consider coverage of *Pseudomonas* and *Enterococcus*. Third- or fourth-generation cephalosporins or carbapenems should be the drug of choice which can be modified later based on blood and urine culture results with susceptibility testing [20]. Our patient grew MDR-ESBL *E. coli *on blood culture which is usually difficult to treat with the usual first-line antibiotics such as third- or fourth-generation cephalosporin and may require later modifications. Choosing the appropriate antibiotics initially plays a major role in predicting mortality. Our patient received meropenem early due to molecular diagnostic testing which gives susceptibility results within hours instead of days. There is no definitive evidence regarding the duration of antibiotic treatment, but most clinicians treat EPN as a complicated urinary tract infection for 7-14 days [4]. Our patient showed clearance of EPN after receiving five days of meropenem although his diagnosis of sepsis with ESBL *E. coli *and subsequent urine leak led to a prolonged antibiotic course. If the response to antibiotics is delayed then, percutaneous nephrostomy tube placement along with urinary stent or radical nephrectomy can be performed. Fluid drained from the percutaneous nephrostomy should be cultured and antibiotics should be modified accordingly [21]. In our patient, ileal conduit urine reflux and ureter stent malposition were the predisposing factors for EPN. Worsening of renal function after appropriate antibiotic treatment and decompression, or a CT scan showing greater than 50% of renal parenchymal destruction with no improvement despite conservative management warrants immediate nephrectomy [22]. The type of nephrectomy depends upon the severity of the disease and the extent of kidney damage. Simple, radical or laparoscopic nephrectomy are the three options currently available. Nephrectomy is always performed if there is no response to conservative treatment but is not performed as first-line management. The mortality rate is approximately 42% if an emergent nephrectomy is required for EPN [23]. Indicators that increase the risk of mortality are altered mental status, thrombocytopenia, severe hyponatremia and renal failure [22]. Therefore, early diagnosis and proactive management can lead to better outcomes in patients with EPN.

## Conclusions

In summary, we report a cancer patient who developed EPN and highlighted other cases in the literature. We analysed that most patients have a genitourinary or gynecologic cancer that leads to urinary flow dysfunction. Secondly, gas-producing pathogens other than *Clostridia* are found which are common causes of urinary tract infections. In addition, our case illustrates the increasing resistance in urinary pathogens that requires second-line antibiotics therapy which requires rapid diagnostic laboratory technology to deliver prompt treatment. Finally, surgical management such as nephrectomy can be avoided in most cases with appropriate antibiotic therapy and urinary decompression procedures such as ureter stents or nephrectomy tube placement.
